# The Design of a Low-Noise, High-Speed Readout-Integrated Circuit for Infrared Focal Plane Arrays

**DOI:** 10.3390/s23218715

**Published:** 2023-10-25

**Authors:** Yusong Mu, Zilong Zhao, Chong Chen, Di Yuan, Jing Wang, Hansong Gao, Yaodan Chi

**Affiliations:** 1 Key Laboratory for Comprehensive Energy Saving of Cold Regions Architecture of Ministry of Education, College of Electronic and Computer, Jilin Jianzhu University, Changchun 130118, China; 2 Changchun Jingyi Optoelectronic Technology Co., Ltd., Changchun 130103, China

**Keywords:** infrared focal plane array, ROIC, capacitor transimpedance amplifier, noise analysis

## Abstract

This paper describes the design of a low-noise, high-speed readout-integrated circuit for use in InGaAs infrared focal plane arrays, and analyzes the working principle and noise index of the pixel circuit in detail. The design fully considers the dynamic range, noise, and power consumption of the pixel circuit in which a capacitance transimpedance amplifier structure is adopted as the input stage circuit, and chip fabrication via an XFAB 0.18 µm CMOS process is successfully realized. The ROIC adopts monolithic integration and implements various functions, such as windowing, subsampling, and different integration and readout modes. The ROIC reached an array scale of 32 × 32, a frame rate of 100 Hz, and a readout rate of 20 Mbps with an analog power consumption of less than 52 mW. The measurement results show that the input reference noise can be reduced to 143 e- via the CDS, and the fully customized scheme has certain advantages in the research of high-performance ROICs.

## 1. Introduction

The InP/InGaAs short-wave infrared single-photon detector (SPAD) is a widely used single-photon detector with relatively mature preparation technology. It can reach its working temperature (of about −40 °C) through a semiconductor thermoelectric refrigeration (TEC) device, which has the advantages of small size, low cost, convenient installation, and portability. In addition, it is easy to realize large-area single-photon arrays using a chip-manufacturing process based on conventional semiconductor diodes. Therefore, infrared thermography has been highly valued since its emergence. Due to its strong anti-interference ability, good concealment, and low level of power consumption, it has many applications in military, aviation, and other fields [1,2,3]. With the continuous development of infrared thermography, the Infrared Focal Plane Array (IRFPA) has gradually gained attention from researchers, especially the 1550 nm InP/InGaAs SPAD array, which is safe for the human eye. At the same time, there is an urgent need for the development of single-photon detectors with high detection efficiencies, high counting rates, high temperatures, and low costs.

At present, crosstalk between the pixels of a focal plane array is the main problem restricting the performance of high-density arrays. In addition, the uniformity of a focal plane array is an important parameter to ensure the operation of the focal plane. The closer the breakdown voltage of pixels in the array, the closer the working voltage is and the easier the working conditions of the array are. Array uniformity includes breakdown voltage uniformity, dark current uniformity, detection efficiency uniformity, and dark count uniformity. It is usually characterized via the breakdown voltage distribution, dark current distribution, dark count distribution, and detection efficiency distribution of the elements of the array. There are many related research reports on this subject.

In 2014, Teledyne Princeton Instruments developed a Geiger-mode focal plane array with single-photon sensitivity [4]. The InP/InGaAs Photo-Diode Array (PDA) is coupled with a custom silicon readout-integrated circuit (ROIC), and the MLA is aligned and integrated onto the back of the PDA. Subsequently, electrical connection with the ROIC was achieved through bonding with ceramic needle insert boards, achieving two specifications, 32 × 32 and 128 × 32, which have pitches of 100 μm and 50 μm, respectively. In 2018, MIT (Massachusetts Institute of Technology, Lincoln Laboratory) developed arrays of custom-fabricated silicon and InP Geiger-mode avalanche photodiode arrays [5]. Starting with 4 × 4 arrays, they recently demonstrated 256 × 256 arrays, and are working to scale up to megapixel-class imagers. In 2015, the China Electronics Technology Group Corporation (in Chongqing) used the photoresist hot-melt method to achieve the monolithic integration of micro-lens arrays on the back of InP substrates and produced InP/InGaAs Geiger-mode focal plane arrays with a size of 8 × 8 and a pixel center-to-center distance of 150 μm [6]. In 2022, the Institute of Semiconductors, CAS, designed a 64 × 64 InP/InGaAs SPAD array, and the pixels in the array adopted a SAGCM structure [7]. Shortly afterwards, the Shanghai Institute of Optics and Fine Mechanics, CAS, developed a prototype of a miniaturized LIDAR system based on the InGaAs Geiger-mode avalanche photodiode array detector developed earlier [8]. The detector used in the prototype was a 64 × 64 InGaAs Geiger APD array, which has a light quantum efficiency of 20% at a wavelength of 1550 nm and a dark counting rate of 20 kHz at low temperatures. 

The history of the development of infrared focal plane array detectors ranges from first-generation products, which mainly focused on scanning imaging with units and utilized multiple devices as cores [9], to the current fourth-generation system-level chip products [10]. At present, SUI, Attollo, Sony, and other companies have developed 5 μm center distance short wave infrared InGaAs focal plane detectors [11]; it can be seen that large-area arrays and high-performance readout circuits have become a trend [12,13,14,15]. Therefore, it is necessary to design a specialized infrared readout circuit to amplify and denoise the signal in order to ensure the effectiveness of signal extraction [16,17,18,19,20]. The authors of [21] used the SFD structure as the pixel unit of the readout circuit, which is simple in structure; the authors of [22] used an amplifier with a correlated double-sampling function to suppress noise; and the authors of [23] simplified the structure of the correlated double-sampling circuit, reducing circuit noise, the power consumption, and the area.

With the continuous improvement and development of the material structure, material quality, preparation process, and quenching circuits of InP/InGaAs short-wave infrared single-photon detectors, the performance of InP/InGaAs SPADs has been significantly improved, and the typical detection efficiency has increased from 20% to 30%, while the dark counting rate has further decreased and is lower than kHz [1]. Another significant performance improvement is the suppression of the reduction in the dead time of the pulse after the device. By optimizing the quenching circuit and using a negative-feedback single-photon detector, the reported dead time has been increased from the micron level to the submicron level [2]. This provides important chip technology support for high-speed applications.

This paper describes the design of a high-dynamic-range, infrared focal-plane-array readout circuit. The pixel adopts the capacitive trans-impedance amplifier (CTIA) circuit type, and a relevant double-sampling circuit is added to reduce the input reference noise and eliminate the interference problem in the process of column bus transmission. The digital circuit adopts a row-by-row and column-scanning method of first selection and then column selection, and proposes a pre-establishment mechanism to improve the reading rate of the circuit and reduce the overall power consumption of the system. Therefore, the characteristics of the high-performance readout circuits in this paper include low noise, large dynamic ranges, low levels of power consumption, capacitors with large storage capacities, and small array sizes and center-to-center distances.

## 2. Infrared Focal Plane Array

### 2.1. IRFPA Architecture

**Figure 1:**
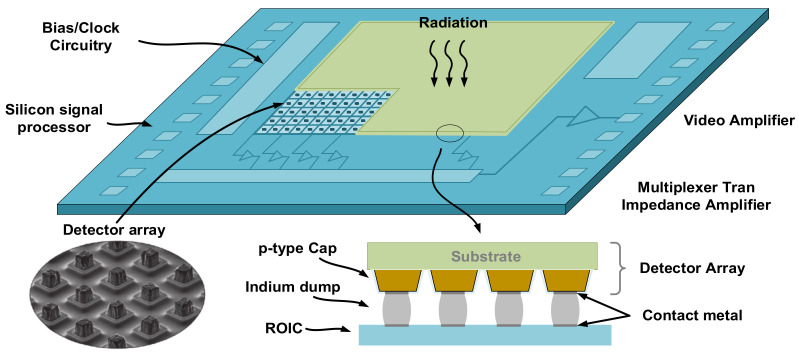
Structural diagram of IRFPA.

Figure 1 is a structural diagram of the IRFPA imaging system, which mainly includes the optical system, IRFPA, ADC, signal processing, data storage, and information display. The IRFPA consists of an infrared detector array and a readout circuit as the core components of the system. The infrared detector arrays are usually composed of N × M detector units, the number of which represents the resolution. The readout circuit has the same area and cell matrix structure as the infrared detector array, and connects its infrared detector array through the Flip-Chip package.

When the IRFPA system receives infrared radiation, the N × M detector units undergo photoelectric conversion, and the readout circuit processes the signal into a voltage signal and outputs it to the outside of the chip. Then, the voltage signal is restored through modules such as ADC and DSP to obtain infrared images at the terminal.

The readout circuit basically adopts a snapshot readout method, whereby all pixels in the pixel array are integrated simultaneously during integration, and then the signal is read out step by step. We performed a time series analysis of the ITR and IWR working modes. The difference between ITR mode and IWR mode relates to whether the signal is integrated and read at the same time, which results in a slower read rate in ITR mode, but less noise interference.

### 2.2. IRFPA on Chip Design

**Figure 2:**
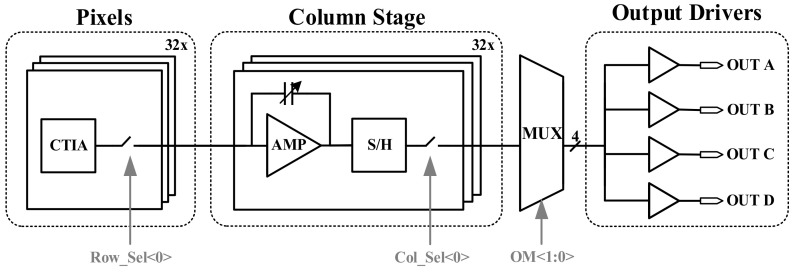
Structural diagram of the analog channels.

Figure 2 is a structural diagram of the analog channels, which mainly include Pixels, the Column Stage, MUX, and Output Drivers. Pixel cells use CTIA to achieve the I-V conversion of signals, and correlated double sampling (CDS) is designed to control noise. In the design of the Column Stage, we use a programmable gain amplifier (PGA) as a column level amplifier to ensure that the signal is fully and accurately amplified to a reasonable range. Further, we design an output buffer with a rail-to-rail amplifier architecture and connect it as a source follower at the back of each output channel.

During a reading cycle, the Row__Sel_ switch will conduct twice. The first time it outputs the integral signal V_int_ from the previous frame to the Column Stage, and the second time it outputs the reset signal V_ref_ from the previous frame to the Column Stage. When processing signals on the Column Stage, V_int_ and V_ref_ are first subtracted using the AMP, and then stored on the capacitor after the S/H. When the Col__Sel_ switch is on, the signal held on the capacitor is selected by the MUX for the corresponding analog channel, and then output to the outside of the chip through the Output Drivers.

This design adopts the telescoped amplifier structure as the main operational amplifier structure in PGA. This design mainly considers the gain and bandwidth characteristics, as the size of the gain determines the accuracy of the circuit, while the bandwidth determines the speed of the circuit. The Output Driver is designed as a circuit structure of a rail-to-rail amplifier, and connected in the form of a source follower to achieve unit gain feedback. Furthermore, the input stage circuit adopts a foldable common source and common gate structure, which can achieve very high input stage gain. The output stage circuit adopts a Class AB structure, which can achieve rail to rail output.

### 2.3. Digital Circuit Design

**Figure 3:**
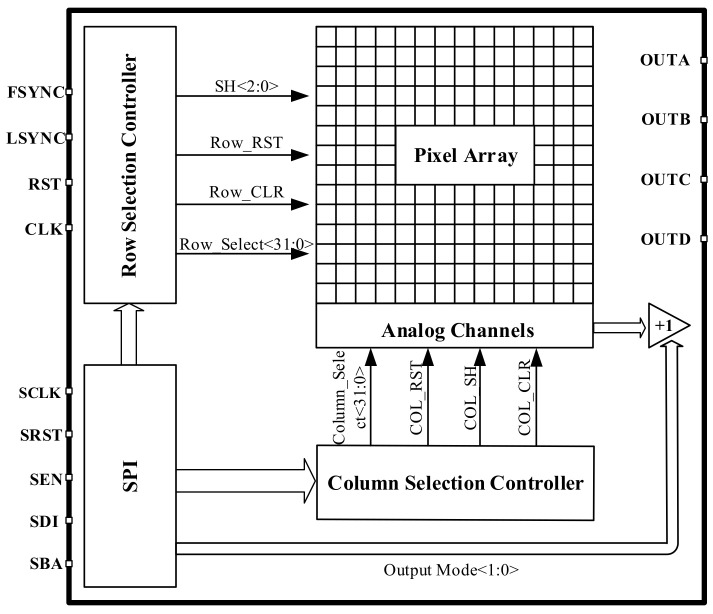
Structural diagram of the digital circuit.

The digital circuit part consists of three modules: serial peripheral interface (SPI), row selection controller and column selection controller. As shown in Figure 3, the readout circuit mainly includes analog channels, output drivers, and bias circuits. The analog channel also includes pixel arrays, column level amplifiers, a multiplexing module, etc. The readout circuit can be compatible with both ITR and IWR integration modes, with an adjustable integration time of 102–109 ns and a time step of 100 ns. We have also designed four high-speed output channels for the readout circuit, which are controlled by digital circuits to achieve three output modes: 1, 2, and 4.

This design requires pixel arrays to have multiple different scanning methods, which can be selected according to the application requirements in different situations. We use the SPI module to serially input configuration information into the chip and control the signal readout of the entire chip. Before each column scan starts, a certain pre-establishment time is given to allow the signal processing module on the column bus to complete the establishment and transmission of signals. 

## 3. CTIA Pixel Circuit

### 3.1. Architecture Design

**Figure 4:**
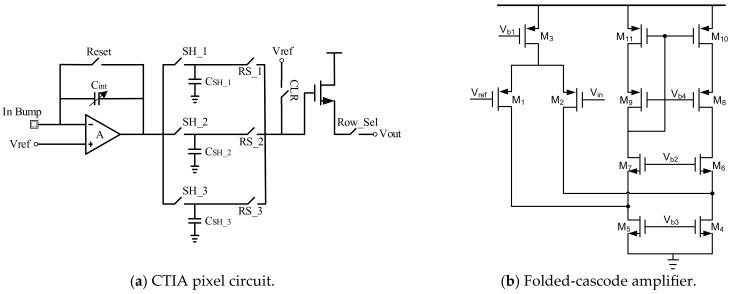
Structural diagram of CTIA: (**a**) pixel circuit in CTIA; (**b**) amplifier structure in CTIA.

This paper improves on the CTIA type circuit to design a low noise, high-speed, and compatible readout circuit with two integration modes. The circuit structure of the pixel circuit is shown in Figure 4a, mainly composed of CTIA, CDS, and a source follower transistor (SF), in addition to two MOS switches. The CTIA is a differential amplifier with matching input-to-tube. The placement and connection of other MOS tubes need to conform to the principle of symmetry. In addition, the pixels are filled with multiple capacitor arrays that have been matched accordingly, each of which is an integer multiple of a single capacitor module and a Dummy configuration to match.

In the CTIA structure, factors such as the performance and area of the amplifier are fully considered. Therefore, we design the folded-cascode amplifier as the most critical amplifier structure. As shown in Figure 4b, the folded-cascode amplifier is designed as a differential structure, which can better eliminate the offset problem at the input end of the operational amplifier and has the advantages of high gain and large output swing. The focus of this design is to reduce the power consumption of the folded cascade amplifier. In the low power cell circuit design, the performance of the amplifier will directly determine the noise and readout accuracy of the circuit.

**Figure 5:**
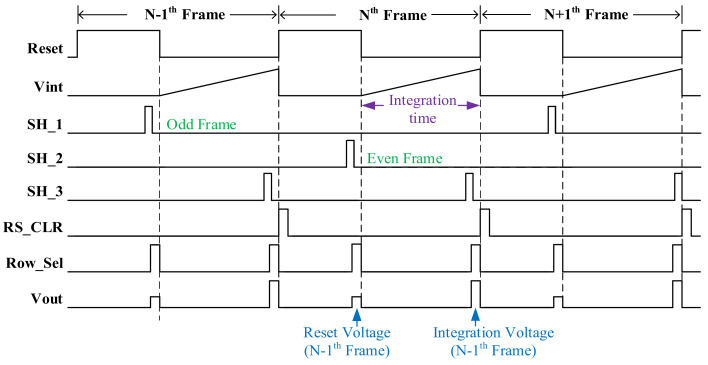
Timing diagram of CDS.

In this paper, the CDS not only eliminates some noise in the circuit, but also needs to be compatible with both ITR and IWR modes. The structure of the CDS consists of three sampling and holding circuits, and each sampling and holding path consists of two switches and an integrated capacitor. Two sampling hold paths are responsible for sampling the reset signals of odd and even frames. The third sampling hold path is responsible for the sampling of the integral signal for each frame. The timing diagram of CDS is shown in Figure 5.

The integral and reset signals are output to the column bus through the SF, and the differential amplifier on the column bus is subtracted within each frame. The on–off CLR eliminates part of the charge stored by the SF input node after the integral signal is read out, which interferes with the reset signal read out later. The SF, which consists of only one MOS tube, is used as a buffer to drive the rear bus in the cell circuit. To reduce power consumption, its offset structure is shared by all cells in each column. The Row__Sel_ switch is turned on and off by a digital sequential generation circuit to control the output signal of the cell to the bus. 

### 3.2. Noise Analysis

The addition of the CDS structure increases the pixel area and power consumption, but it can eliminate the reset noise within the cell, which greatly simplifies the complexity of the circuit in the column bus and avoids the interference of the signal between the column buses. Although the reset noise of the circuit can be eliminated by adding the CDS inside the pixel, it will also introduce its own KTC noise. Therefore, it is significant to analyze the noise of the circuit and compare the effects of CDS noise and reset noise on the circuit.

There are two main noise sources in CTIA circuit, the thermal noise of the reset switch and the thermal noise of the amplifier. Since these two noise sources are not related, we can consider them separately and then sum the results in RMS.

**Figure 6:**
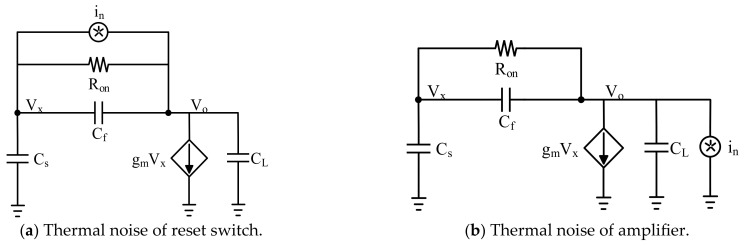
Equivalent circuit diagram of noise: (**a**) switch tube thermal noise small signal equivalent circuit; (**b**) amplifier thermal noise small signal equivalent circuit.

As shown in Figure 6a, assuming that the output impedance of the amplifier is high enough, two equations can be derived from the simplified small signal equivalent circuit:

(1) $$v_{x}C_{x}s+\left(v_{x}-v_{o}\right)\left(C_{f}s+\frac{1}{R_{on}}\right)+i_{n}=0$$

(2) $$g_{m}v_{x}+C_{L}sv_{o}+\left(v_{o}-v_{x}\right)\left(C_{f}s+\frac{1}{R_{on}}\right)-i_{n}=0$$

Thus, we can get the transfer function of thermal noise and the total charge of the switch tube:

(3) $$H_{n}\left(s\right)=\frac{R_{on}g_{m}C_{f}+s\left(C_{s}C_{f}+C_{f}C_{L}+C_{s}C_{L}\right)}{s^{2}R_{on}\left(C_{f}C_{s}+C_{f}C_{L}+C_{s}C_{L}\right)+s\left(R_{on}g_{m}C_{f}+C_{f}+C_{s}\right)+g_{m}}$$

(4) $$S_{n}\left(f\right)=\frac{4kT}{R_{on}}$$

In this paper, the grid width W of the reset tube is made as small as possible to eliminate the influence of clock feed and charge injection, and the grid length L of the reset switch tube is made larger to avoid the charge leakage of the switch. These designs result in a relatively high *R_on_* and a smaller *g*. Therefore, we can assume *R_on_* = 1/*g_m_* to integrate the noise and get the switch noise, as

(5) $$\bar{{q_{n}}^{2}}=\int_{0}^{\infty }S_{n}\left(f\right){\left|H_{n}\left(s\right)\right|}^{2}df=kT\left(C_{f}+\frac{C_{f}C_{s}}{C_{f}+C_{L}+C_{s}}\right)$$

Similarly, according to the small signal equivalent circuit shown in Figure 6b, the charge of amplifier thermal noise can be expressed as

(6) $$\bar{{q_{a}}^{2}}=\int_{0}^{\infty }S_{a}\left(f\right){\left|H_{a}\left(s\right)\right|}^{2}df=kT\gamma \alpha \left(\frac{C_{s}}{C_{f}+C_{L}+C_{s}}\right)$$

In general, the value of the *γ* generally ranges from 1 to 1.5, and the contribution of load to noise is expressed as $\alpha =\left(1+\frac{g_{mp}}{g_{mn}}\right)$. The total reset noise can be expressed as

(7) $$\sigma_{RST}=\sqrt{kT\left(C_{f}+C_{s}\left(C_{L}+2\alpha C_{s}\right)/C_{tot}\right)}/q$$

In the above formula, the total noise consists of three parts. The noise of the first part is the noise of the feedback capacitance. The second part of the noise is the KTC noise of the input capacitance and the load capacitance. The third part of the noise is the amplifier noise. Furthermore, $C_{tot}=C_{s}+C_{f}+C_{L}$, $\alpha =2\left(1+g_{m7}/g_{m1}\right)/3$, where *g*_*m*1_ and *g*_*m*7_ refer to input transistors and load crystals in operational amplifiers. In addition, we also consider the noise caused by the thermal noise of amplifiers, *C_SH_* and SF, using the above method. All noise expressions are shown in Table 1.

In the above formula, CSH is the capacitance value maintained for sampling, and SF is the source follower. $C_{x}=C_{f}C_{s}+C_{s}C_{L}+C_{L}C_{f}$. The calculation results for each noise can be obtained from the above formulas as shown in the table with the integrated capacitance of 30 fF. The final calculation shows that CDS can reduce the cell noise by about 1/3. By comparing the readout noise before and after adding the CDS structure, it was found that adding the CDS structure inside the pixel can effectively reduce readout circuit noise, providing a theoretical basis for circuit design.

## 4. Readout Integrated Circuit for IRFPA

### 4.1. ROIC on the Chip

The ROIC circuit is integrated on a single chip. In addition to the analog signal channel, the digital control part and bias circuit are integrated on the chip. This design adopts the XFAB 1P4M 0.18 μm CMOS process with an overall area of 2.2 mm × 1.6 mm. We try to avoid mutual interference between the analog circuit and the digital circuit in the design of the ROIC. The analog circuit includes the signal channel, output driver and bias generation circuit. 

**Figure 7:**
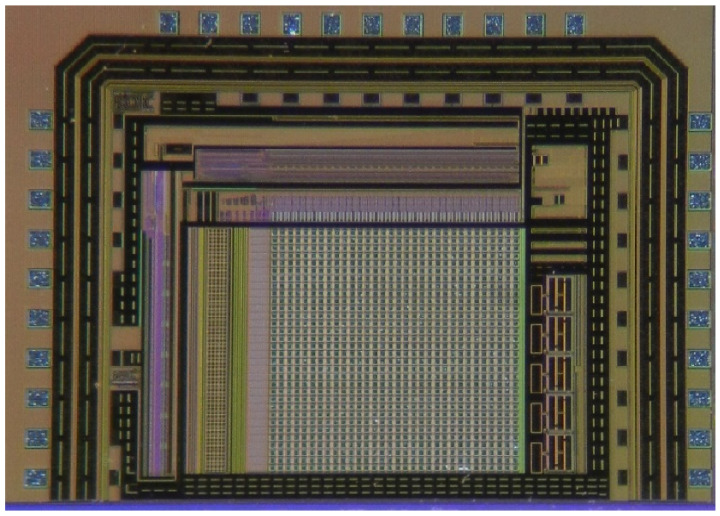
Photos of the complete chip.

Figure 7 is the real chip of ROIC, which contains all the core circuit layout, the PAD interface and the Sealring. In this design, the I/O ring outside the chip is disconnected, but it also conforms to the electrical design rules. The signal channel includes the pixel array, column level amplifier, sample and hold, multiplexing, and other modules. The most important thing in the layout design of analog circuits is the layout and wiring, especially the placement and connection of MOS tubes and capacitors in the unit pixel with limited area. The digital circuit mainly includes three parts: row selection control, column selection control, serial peripheral interface and other modules. The layout design of digital circuits has strict requirements for wiring, and the length, width, interconnection mode, etc., of lines will affect the performance of circuits.

In this article, the infrared detector array of IRFPA is made of InGaAs material, which can accept a reverse bias voltage greater than 10 V in the wavelength range of 0.9–1.7 μm, and the dark current in dark environments is 10-20 nA. The readout circuit adopts a single-chip integrated approach, balancing the advantages and disadvantages of factors such as unit pixel area, power consumption, and performance. As shown in Table 2, the ROIC pixel array has a 32 × 32 frame rate and a frame frequency of 100 Hz. It supports one, two, and four output modes. The read rate is 20 Mbps.

### 4.2. Results and Analysis

**Figure 8:**
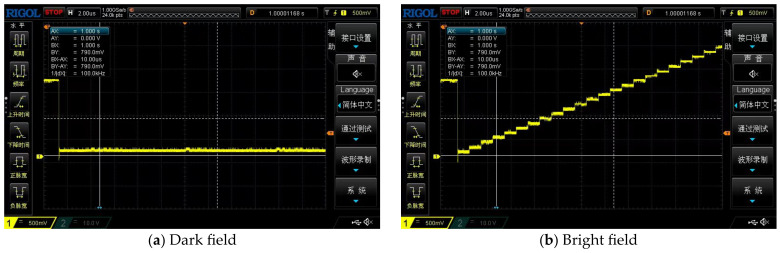
The waveform of the output signal: (**a**) output signal in dark field; (**b**) output signal in bright field.

We performed the functional verification of the noise of the designed chip used in the MV-IS evaluation system, which can provide professional light source measurement and a darkroom environment. According to the definition of signal to noise (SNR) in EMVA Standard 1288 [24], we conducted the light source measurement and darkroom environment measurement, respectively, and verified that the chip has the overall functionality intended. Figure 8 shows the oscilloscope waveform of a group of measurement results under dark field and bright field environments.

**Figure 9:**
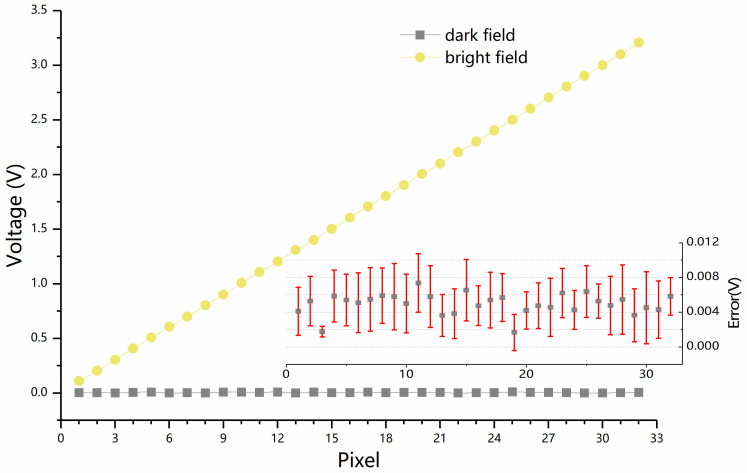
Noise results of ROIC output signal under dark field and bright field conditions.

Further, we convert the analog signal output by the ROIC into digital signal, and then transmit the digital signal to the computer through the logic analyzer for data processing. The processed results are shown in Figure 9, where the abscissa is thirty-two pixels in the line array, and the ordinate is the voltage value read by the readout circuit. The analysis shows that the noise error in the dark field environment is within a reasonable range, less than 20 mV. At the same time, the error of the noise in the bright field is also of this order of magnitude.

### 4.3. Discussion

In this paper, noise is a key performance parameter in pixel circuit design, and detailed model analyses and theoretical calculations have been carried out in the previous chapter. Under normal working conditions, noise will interfere with the signal at certain time intervals, and the sizes of these noises are random. Therefore, we have collected multiple noise data and drawn a histogram of noise distribution. 

**Figure 10:**
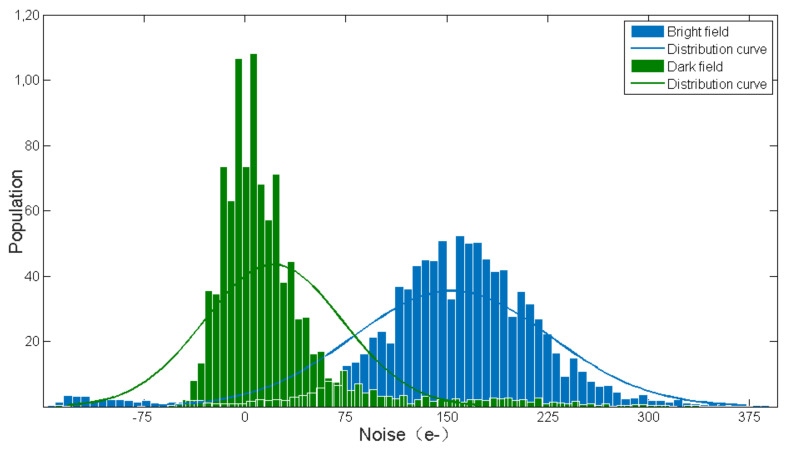
Histogram of noise distribution.

Figure 10 is the noise distribution histogram obtained after 1000 noise tests. The results are basically in line with the Gaussian distribution, the mean of pixel noise in the bright field is 143 e-, and the mean of pixel noise in the dark field can be ignored. Compared with the previous theoretical calculations, we find that the measured noise is smaller than that which was theoretically calculated. The reason for the above result is the influence of parasitic parameters caused by layout, which leads to noise suppression to a certain extent. However, the test results under dark field conditions exclude the noise impact of the detector, indicating that the noise of the ROIC part is completely limited. 

Therefore, the main indicators of ROIC in this paper, such as noise, consumption, and pixel rate, have been compared horizontally with those in the existing literature, as shown in Table 3.

In terms of noise indicators, the small area array test results in this article have significant advantages compared to those in the literature (in 2016). In addition, the larger pixel array areas shown in references [14] (in 2021) and [15] (in 2023) are not on the same order of magnitude as the noise in this paper, but compared to the noise converted into a single pixel, this paper’s findings are superior to those of [14] and similar to [15]. In terms of other indicators, as Refs. [14,15] show large area arrays, they cannot be compared intuitively in terms of pixel rate and consumption. Generally, a larger array area will increase noise and consumption, while reducing rate. In Table 3, we see that the input circuits of DI and IR are, respectively, used in Refs. [14,15], and the 55 nm process in [15] is also greatly improved compared with other studies. Considering all performances, the ROIC used in this paper improves the performance in terms of pixel rate, consumption and noise to the greatest extent, and has obvious advantages compared with studies [14,19], while it yields results similar to those of [15].

## 5. Conclusions

In this manuscript, we proposed a low-noise, high-speed readout-integrated circuit for InGaAs infrared focal plane array. Its main feature is that the design fully considers the dynamic range, noise and consumption of pixel circuit. Therefore, the performance of other aspects of the chip is not over-designed to make sure its meets the application requirements at minimum cost. Chip manufacturing via the XFAB 0.18 µm CMOS process is successfully realized, and the fully customized scheme has certain advantages in the research of high-performance ROIC. The pixel array size is 32 × 32, with a frame rate up to 100 Hz, supporting one-way, two-way and four-way output modes, and with a readout rate of 20 Mbps and an overall chip area of 2.2 mm × 1.6 mm. The pixel input stage of ROIC uses a CTIA structure, which can be used in the three states of very high gain (VHG), high gain (HG) and low gain (LG), respectively. In the process of full custom design, we try to avoid mutual interference between the two parts. Finally, the test results of the chip prove that it achieves good performance in terms of noise, readout rate and power consumption.

## Figures and Tables

**Table 1:** Pixel circuit readout noise calculation results.

Noise Type	Expression	Noise/e- (No CDS)	Noise/e- (Including CDS)
Switch reset	$\sigma_{RST}=\sqrt{kT\left(C_{f}+C_{s}\left(C_{L}+2\alpha C_{s}\right)/C_{tot}\right)}/q$	184	0
Thermal of amplifiers	σAMP=Cf+Csq2αkTCsCx	97	138
Sample and hold	$\sigma_{SH}=\frac{C_{f}}{q}\sqrt{\frac{kT}{C_{SH}}}$	24	34
Source follower	$\sigma_{SF}=\frac{C_{f}}{q}\sqrt{\frac{2}{{\pi R_{out}C}_{out}}\times \frac{4kT}{g_{m,SF}}}$	13	13
Total pixel	$\sigma_{ROIC}=\sqrt{{{{\sigma_{RST}}^{2}+\sigma }_{AMP}}^{2}+{\sigma_{SH}}^{2}{+\sigma_{SF}}^{2}}$	210	143

**Table 2:** Design indicators for chips.

Function	Parameter
Detector array	32 × 32
Unit area	25 × 25 μm
Mode	ITR and IWR
Output channel	1, 2, 4 Optional
Frame frequency	>100 fps
Clock frequency	5 MHz
Consumption	200 mW
Operating voltage	3.3 V(analog); 1.8 V(digital)

**Table 3:** ROIC performance comparison.

Design	This Work	2016 [19]	2021 [14]	2023 [15]
Technology	0.18 μm	0.18 μm	0.5 μm	55 nm
Input Circuit	CTIA	CTIA	DI	IR
Pixel Array	32 × 32	4 × 4	640 × 512	8192 × 8192
Pixel Pitch	25 μm	15 μm	25 μm	10 μm
Pixel Rate	20 MHz	3 MHz	-	0.5 MHz
Output Channel	1, 2, 4 Optional	-	1, 2, 4 Optional	-
Frame Rate	>100 fps	100	60	80
Input Noise	143 e-	180 e-	18 Me-	6 Me-
Supply Power	3.3 V	-	5.5 V	3.3 V
Consumption	52 mW	-	-	16.5 uW

## Data Availability

Not applicable.
